# Efficient Gene Editing for Heart Disease via ELIP-Based CRISPR Delivery System

**DOI:** 10.3390/pharmaceutics16030343

**Published:** 2024-02-29

**Authors:** Xing Yin, Romain Harmancey, Brion Frierson, Jean G. Wu, Melanie R. Moody, David D. McPherson, Shao-Ling Huang

**Affiliations:** 1 Division of Cardiology, Department of Internal Medicine, McGovern Medical School, The University of Texas Health Science Center at Houston, Houston, TX 77030, USA; romain.harmancey@uth.tmc.edu (R.H.); brion.frierson@uth.tmc.edu (B.F.); melanie.r.moody@uth.tmc.edu (M.R.M.); david.d.mcpherson@uth.tmc.edu (D.D.M.); 2 Department of Diagnostic Sciences, The University of Texas Health Science Center at Houston, Houston, TX 77054, USA; jean.g.wu@uth.tmc.edu

**Keywords:** echogenic liposomes (ELIP), CRISPR, single-guide RNA, gene editing, gene delivery

## Abstract

Liposomes as carriers for CRISPR/Cas9 complexes represent an attractive approach for cardiovascular gene therapy. A critical barrier to this approach remains the efficient delivery of CRISPR-based genetic materials into cardiomyocytes. Echogenic liposomes (ELIP) containing a fluorescein isothiocyanate-labeled decoy oligodeoxynucleotide against nuclear factor kappa B (ELIP-NF-κB-FITC) were used both in vitro on mouse neonatal ventricular myocytes and in vivo on rat hearts to assess gene delivery efficacy with or without ultrasound. In vitro analysis was then repeated with ELIP containing Cas9-sg-IL1RL1 (interleukin 1 receptor-like 1) RNA to determine the efficiency of gene knockdown. ELIP-NF-κB-FITC without ultrasound showed limited gene delivery in vitro and in vivo, but ultrasound combined with ELIP notably improved penetration into heart cells and tissues. When ELIP was used to deliver Cas9-sg-IL1RL1 RNA, gene editing was successful and enhanced by ultrasound. This innovative approach shows promise for heart disease gene therapy using CRISPR technology.

## 1. Introduction

Recent advancements in CRISPR-based gene editing technologies have renewed interest in gene therapy for the treatment of cardiovascular disease [1,2,3,4,5,6]. A pivotal factor for the success of cardiac gene therapy involves designing a biodistribution strategy that can effectively deliver genetic materials into target cells to achieve the desired effect. Clinical and preclinical investigations, both in vitro and in vivo, have consistently pointed to liposomes as a promising delivery platform for a wide range of therapeutic applications. This is primarily due to their exceptional biodegradability and biocompatibility as carriers for CRISPR (Clustered Regularly Interspaced Short Palindromic Repeats)-based gene editing systems [4,7,8]. Despite these advantages, several challenges persist in combining liposomes with CRISPR-based technology for gene therapy, particularly regarding the efficacy and targeting capabilities of the approach. A notable drawback of the strategy remains the lack of cardiac tissue permeability to liposomes, which limits the delivery of cargo to cardiomyocytes.

Ultrasound energy can increase cell membrane permeability to facilitate the intracellular delivery of bioactive molecules and nucleic acids, thus leading to enhanced transfection efficiency in vitro and in vivo by cavitation [9,10,11]. We have developed a gas-containing liposomal contrast agent (echogenic liposome or ELIP) that enhances therapeutic loading and tissue penetration upon ultrasound application [9,10,11,12,13]. Our methodology allows for the generation of customizable, ultrasound-responsive liposomes whose lipid composition and acoustic sensitivity can be adjusted to achieve ideal ultrasound properties for enhanced tissue delivery and long-term stability, making them potentially suitable for clinical applications. Our previous studies have shown the remarkable capability of lipids, prepared using a process involving hydration, sonication, and freeze-drying/thawing in the presence of mannitol, to efficiently encapsulate a significant portion of the aqueous phase oligodeoxynucleotides (ODNs). Entrapment of genetic materials in acoustically responsive liposomes can potentially be further enhanced by adjusting ultrasound parameters during the encapsulation process. Moreover, cargo uptake at the disease delivery site can also be improved using ultrasound to enhance penetration [12,13]. The presence of highly reflective and stable gas pockets within the lipid bilayer contributes to the required ultrasound reflectivity [14]. This ELIP delivery system has already demonstrated strong efficacy and selectivity in delivering therapeutic agents for the treatment of atherosclerosis and other cerebrovascular diseases [9,11]. Stimuli-responsive liposomes are now being explored as a promising way to release the CRISPR complex in response to ultrasound exposure [15,16].

In this study, we tested the utilization of the ELIP carrier for the delivery of nucleic acids and CRISPR-based genetic materials, specifically in cardiomyocytes. Using a fluorescein isothiocyanate-labeled decoy oligodeoxynucleotide against nuclear factor kappa B (ELIP-NF-κB-FITC) and a Cas9-sg-IL1RL1 (interleukin 1 receptor-like 1) RNA as proof of concept, our ELIP carrier provided increased transfection and gene editing efficiencies when combined with ultrasound treatment, both in vitro in isolated mouse neonatal ventricular myocytes and in vivo in the adult rat heart. Hence, this novel platform has demonstrated its ability to site-specifically and efficiently deliver genes into cardiac cells and tissues, thus showing promise in targeted gene therapy for heart diseases. Such a method may offer a non-invasive and efficient means to deliver genetic materials to disease sites, both in vitro and in vivo, for diverse applications in research, biotechnology, and medicine.

## 2. Materials and Methods

1,2-dipalmitoyl-snglycero-3-phosphocholine (DPPC), 1,2-dipalmitoyl-sn-glycero-3-[phospho-rac-1-glycerol] (DPPG), 1,2-dioleoyl-sn-glycero-3-phosphoethanolamine (DOPE), 1,2-dioleoyl-3-dimethylammonium-propane (DODAP), 1,2-dioctadecanoyl-sn-glycero-3-phosphocholine (DSPC), 1,2-stearoyl-sn-glycero-3-phosphoethanolamine-N-[methoxy(polyethylene glycol)-2000] (ammonium salt) (18:0 PEG2000 PE), and cholesterol (CH) were purchased from Avanti Polar Lipids (Alabaster, AL, USA) and stored at −20 °C in chloroform. (6Z,9Z,28Z,31Z)-Heptatriaconta-6,9,28,31-tetraen-19-yl 4-(dimethylamino) butanoate (DLin-MC3-DMA) was purchased from MedKoo (MC3; MedKoo Biosciences, cat. no. 555308). FITC-NF-κB decoy sense ODN (5′-AGTTGAGGGGACTTTCCCAGGC/36-FAM/-3′) and Iowa BlackTM FQ labeled NF-κB decoy antisense ODN (5′-/5IAbFQ/GCCTGGGAAAGTCCCCTCAAC T-3′) were synthesized using Integrated DNA Technologies, Inc. (Coralville, IA, USA). Mannitol was purchased from Sigma Chemical Co. (St. Louis, MO, USA). TrueCut Cas9 Protein V2 and Opti-MEM™ I Reduced Serum Medium were purchased from Life Technologies Corporation (Carlsbad, CA, USA). Candidate sgRNAs were purchased from Synthego Corporation (Redwood City, CA, USA). Primers targeting IL1RL1 for PCR analysis were purchased from IDT (Integrated DNA Technologies, IDT). All chemicals were purchased from Sigma-Aldrich (St. Louis, MO, USA) unless otherwise stated.

### 2.1. Preparation of Echogenic Liposomes (ELIP)

The selection of lipids and their ratios for ELIP involves lipid composition, considering favorable biocompatibility and stability by incorporating cholesterol for membrane stability. It extends to liposome size, affecting tissue penetration and circulation times, surface charge impacting stability and interactions, drug encapsulation/release dynamics, stability, and storage conditions to prevent issues such as aggregation, leakage, and degradation. Furthermore, a focus on biocompatibility is crucial to minimize adverse effects, and experimental optimization is achieved by evaluating parameters under diverse conditions. ELIP was prepared following previously published methods with modifications [12]. Lipids were mixed in chloroform at specific molar ratios, as indicated below. Initially, 5 mg of lipids were combined with chloroform (9:1). Subsequently, the solvent was removed using argon gas in a 50 °C water bath, forming a thin film in a glass vial. This film was vacuumed for 4–6 h to eliminate all solvents. The dried lipid film was hydrated with 500 μL of deionized water (nuclease-free water for RNA) and warmed up to dissolve completely, subsequently was sonicated until super clear in the bath sonicator for a short time before adding the same volume of 0.32 M D-Mannitol (deoxygenated mannitol). The created liposomes were transferred into a sealed 2-mL glass vial with a Teflon-rubber septum cap. Using a 12-mL syringe attached to a “27 G × ½” needle, 10-mL of octafluoropropane (OFP) gas was injected into the glass vial through the Teflon-rubber septum. The gas-pressurized liposomal dispersion was frozen at −70 °C and then thawed by releasing the pressure in the vial upon removing the cap. After thawing, the loading, release profile, and delivery characteristics of echogenic liposomes (ELIPs) were studied.

### 2.2. Preparation of ELIP-Containing NF-κB-FITC (ELIP-NF-κB-FITC)

FITC (green fluorescence) labeled NF-κB oligonucleotide (ODN) was designated NF-κB-FITC. Lipids were mixed in chloroform with a specific composition consisting of DPPC/DOPE/DPPG/cholesterol (36:36:8:20, mole ratio). ELIP containing NF-κB-FITC was prepared using a conventional procedure of hydrating the lipid film, sonication, freezing, and drying, following the procedure described earlier.

### 2.3. Preparation of ELIP for CRISPR Complex

Lipids were mixed in chloroform with a specific composition consisting of MC3/DODAP/DSPC/cholesterol/PEG2000 PE (45:10:9:34.6:1.4, mole ratio). ELIP for the CRISPR complex was prepared using a conventional procedure of hydrating the lipid film, sonication, freezing, and drying, following the procedure described earlier.

### 2.4. Characterization of Loading Efficiency and Ultrasound-Triggered Release

Loading efficiency and ultrasound-triggered release were quantified using the NanoDrop method, as previously described [17,18]. For the loading efficiency, ELIP without encapsulants was used to determine background noise. At each sampling time, ELIP were loaded with an aqueous solution containing Cas9 and sg-IL1RL1 RNA (Ctotal) at a 1:1protein/gRNA mole ratio with or without exposure to ultrasound (1 MHz, 1 W/cm^2^, 100%, 15 s). Subsequently, the particles were subjected to centrifugation at 15,000× *g* for 15 min, with careful collection of the supernatant for quantification of unencapsulated Cas9 protein and sgRNA. The concentrations of unencapsulated Cas9 and sgRNA (C1) were measured with a NanoDrop One spectrophotometer.

**Figure 1:**
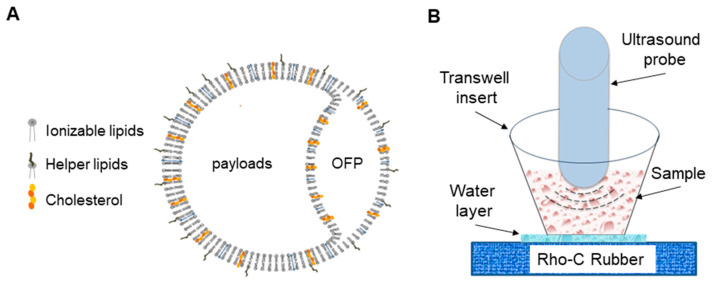
Proposed structure of gas-containing echogenic liposomes (ELIP) and experimental setup for ELIP applications. (**A**) The ELIP loaded with payloads is typically composed of three main lipid components, including ionizable cationic/anionic lipids, helper lipids, and cholesterol. (**B**) In vitro, the setup comprises a Costar transwell insert with a 0.4 μm pore polyester membrane placed on Rho-C rubber. The polyester membrane enables 100% ultrasound wave transmission, while the Rho-C rubber prevents wave reflection. To prevent air interference, a water layer separates the membrane and rubber.

For ultrasound-triggered release experiments, cell culture inserts with 0.4 μm pore membrane-lined bottoms (Costar Corning Inc., Corning, NY, USA) positioned on top of a Rho-C rubber pad were employed (Figure 1). A calibrated 1-MHz continuous wave ultrasound transducer (Sonitron 1000, Rich-Mar Corp, Inola, OK, USA), positioned 5 mm above the insert’s membrane, was used to trigger the release of the ELIP cargo (1 MHz, 2 W/cm^2^, 100%, 120 s). Following exposure to ultrasound, the particle suspension underwent centrifugation at 15,000× *g* for 15 min. The supernatant was carefully collected, and unencapsulated Cas9 and sgRNA (C2) were quantified with a NanoDrop One spectrophotometer. Then, we determined the respective concentrations of encapsulated Cas9 and sgRNA, referencing a calibration curve established with empty ELIP containing similar lipid profiles. Different concentrations of Cas9 and sgRNA diluted in solvents, either nuclease-free water or Opti-MEM reduced serum medium (Thermo Fisher Scientific), depending on the experiment, were added into these liposomes for calibration (in duplicate), as used to prepare the sample protein/RNA.

The actual loading percentage (%) and ultrasound-triggered release percentage (%) for Cas9-sgRNA RNP were calculated using the following formulas:

Loading (%) = 100 × (Ctotal − C1)/Ctotal(1)

Ultrasound-triggered additional release (%) = 100 × (C2 − C1)/Ctotal(2)

### 2.5. Isolation and Culture of Primary Ventricular Myocytes

All animal studies were approved by the University of Texas Health Science Center in Houston’s Animal Welfare Committee. Cardiomyocytes from 1-day-old C57BL/6J mice were isolated and cultured as described previously [19]. Mice were euthanized, and hearts were harvested and placed in ice-cold Hanks’ solution containing 5 mM HEPES (pH 7.4) and 100 U/mL penicillin-streptomycin (Thermo Fisher Scientific, Waltham, MA, USA). After the atria were removed, the ventricles were sliced into small pieces (2–4 pieces per heart) and washed three times using cold fresh Hanks’ solution. The tissue was digested using fresh Hanks’ solution containing 1 mg/mL collagenase type II (Worthington Biochemicals. Lakewood, NJ, USA) and subjected to gentle rocking for 2 hours at room temperature. Suspended cells were pelleted using centrifugation at 800× *g* for 5 min at 4 °C. The dissociated cells were cultured with Dulbecco’s Modified Eagle’s Medium (DMEM; Invitrogen, Waltham, MA, USA, Life Technologies, Carlsbad, CA, USA) containing 10% fetal bovine serum (Invitrogen, Life Technologies), L-glutamine (2 mM, Invitrogen, Life Technologies), 5 mM HEPES (pH 7.4), and 100 U/mL penicillin-streptomycin (Invitrogen, Life Technologies) in a humidified atmosphere with 5% CO_2_ for 1 h at 37 °C to allow non-cardiomyocytes to attach to the bottom of the culture dishes. The culture medium containing the enriched cardiomyocyte fraction in suspension was then transferred into 6-well plates, and cells were allowed to proliferate in a humidified atmosphere and 5% CO_2_ at 37 °C. Once confluent monolayers were formed, cells were rendered quiescent by culture in serum-free DMEM for 48 h.

### 2.6. Treatment of Cultured Mouse Neonatal Cardiomyocytes

The cultured cells were cultured, split, and then suspended with the growth medium. The cells were transfected/treated with ELIP-NF-κB-FITC (final concentration 10 nM) or ELIP-Cas9/sg-IL1RL1 RNA RNP (final concentration 4 μg/mL) separately by manipulating the ultrasound (US) parameters (1 MHz, 2 W/cm^2^, 100%, 120 s), then seeded onto culture plates. In order to determine uptake efficiency, the cells were seeded onto 96-well tissue culture plates and allowed to grow for 48 h post-treatment. Next, the cells were washed with PBS and lysed by adding 100 μL of a 90% DMSO: 10% PBS solution (v:v) for 10 min in the dark and at room temperature under gentle shaking. The fluorescence intensity in each well was measured using a Synergy microplate reader with fluorescence excitation and emission wavelengths set at 490 and 520 nm, respectively.

For fluorescence imaging, the treated cells were cultured on culture slides overnight before being fixed with 3.7% paraformaldehyde in PBS (pH 7.4) for 20 min at room temperature, permeabilized with 0.3% Triton X-100 in PBS for 5 min, and incubated in blocking solution consisting of 10% bovine serum albumin in PBS for 2 h. Next, the cells were washed three times for 10 min with PBS, followed by a 3 min incubation with DAPI for nuclear staining. The fluorescent intensities were quantified using NIH ImageJ software version 1.54.

In order to determine the efficiency of gene editing with the ELIP-CRISPR complex, the cardiomyocytes were transfected with ELIP-Cas9/sg-IL1RL1 RNA RNP following Invitrogen’s TrueCut Cas9 Protein V2 protocol. The RNP loading was achieved using a molar ratio of ELIP/CRISPR/Cas9 protein/the gRNA of 400:1:1 in Opti-MEM (Thermo Fisher Scientific). The cardiomyocytes were seeded onto 24-well culture plates and incubated at 37 °C for 72 h after treatment.

### 2.7. In Vivo Delivery of ELIP-FITC-Labeled Oligonucleotides

Male Sprague Dawley rats (250–350 g) were anesthetized and administered 200 μL of a 3 μmol/L solution of ELIP-NF-κB decoy sense via left ventricular injection under aortic clamping and 5 min ultrasound exposure (Duty cycle 100%, frequency 1 MHz, Intensity 2 W/cm^2^, Transducer 5 cm^2^). Next, the hearts were excised, rinsed with PBS, and fixed in a 4% paraformaldehyde solution (PFA) for 24 h. Hearts were subsequently transferred in a 30% sucrose solution and stored at 40 °C for 48 h. Hearts were then embedded in an OCT compound and allowed to freeze at −20 °C for 4–6 h prior to being stored at −80 °C. The whole hearts were sectioned at a thickness of 25 μm and imaged using fluorescence microscopy. Fluorescent intensities were measured and quantified using NIH ImageJ software version 1.54.

### 2.8. Determination of Gene Editing Efficiency

To assess gene editing efficiency at the target genomic DNA sites (Table 1), neonatal cardiomyocytes were transfected with Cas9 and sgRNAs via ELIP, following the modified user manual for Invitrogen’s TrueCut Cas9 Protein V2. Briefly, on the day of transfection, cells were detached via trypsinization, resuspended in an appropriate volume of growth medium, and then counted and checked for viability. The appropriate number of cells was transferred into a 15-mL centrifuge tube, then pelleted by centrifugation at 100–400× *g* for 5 min at room temperature. The cell pellet was then resuspended in the culture medium at the desired amount. Cell Transfection was carried out using cell culture inserts with membrane-lined bottoms and a Rho-C rubber pad, as described above. The ELIP (1 mg/mL) was used in place of the Lipofectamine™ CRISPRMAX™ reagent. Cells were transfected with or without ELIP-Cas9/sg-IL1RL1 RNA (ultrasound parameters: Duty cycle 100%, frequency 1 MHz, Intensity 2 W, Duration 120 s, Transducer 5 cm^2^). Then, following Invitrogen’s user manual, the cells were seeded onto 24-well tissue culture plates at a density of 100,000 cells per well and cultured for 72 h. Subsequently, following the GeneArt Genomic Cleavage Detection kit manufacturer’s instructions (Thermo Fisher Scientific), genomic DNA from transfected cells was isolated, and the IL1RL1 locus where gene-specific double-strand breaks occurred was amplified by PCR using specific primers (Table 2). The resulting PCR product underwent denaturation and re-annealing, generating mismatches between strands with indels. Following enzymatic digestion, the resulting PCR products were resolved on a 2% TAE-agarose gel stained with ethidium bromide, and gel images were analyzed using ImageJ software version 1.54. The following equation was used to calculate the cleavage efficiency:

Cleavage Efficiency= 1 − [(1 − fraction cleaved)½](3)

Fraction Cleaved= sum of cleaved band intensities/(sum of the cleaved and parental band intensities)(4)

The cleavage efficiency for the control template and primers was calculated as [sum of cleaved band intensities/(sum of cleaved and parental band intensities)] × 100%(5)

### 2.9. Design of gRNA and Primers

The design of single guide RNAs (sgRNAs) targeting IL1RL1 was conducted by employing previously published sequences (NM_001025602.4). We then employed Synthego’s guide design website (https://www.synthego.com/products/bioinformatics/crispr-design-tool (accessed on 30 March 2023) to screen and select the top 4 recommended guides. Primers targeting IL1RL1 for PCR analysis were purchased from IDT (Integrated DNA Technologies, IDT). Evaluation of candidate sgRNAs was performed in neonatal cardiomyocytes using Invitrogen’s user manual.

### 2.10. Statistical Analysis

Data were analyzed using Microsoft Excel and GraphPad Prism 5.0. All values are expressed as means with standard derivation (S.D.) with *n* = 5 independent biological replicates in each group. For comparison between multiple groups, the Kruskal–Wallis analysis of variance (ANOVA) was performed, followed by post hoc multiple comparisons of mean ranks for all groups. *p*-values less than 0.05 were considered significant. Data were presented as mean ± S.D.

## 3. Results

### 3.1. Design and Preparation of Gas-Containing Ionizable Liposomes and Experimental Setup

Our goal was to optimize ultrasound-responsive gas-containing echogenic liposomes loaded with genetic materials for advancing gene therapy. Previous studies have used anionic lipid formulations to make echogenic liposomes and demonstrated gas trapped between their bilayers [11,12,16,20,21,22,23,24,25]. For gene delivery, in addition to using the previously published formulation for ODN delivery, we modified the formulation with cationic lipids for CRISPR delivery. Based on the ultrasound imaging and the previous studies above, the fundamental structure of the liposome was hypothesized, as illustrated (Figure 1A). As shown in Figure 1B, an experimental setup using ELIP was employed for in vitro cell experiments, with specific procedural information detailed in the Section 2.

### 3.2. Octafluoropropane Enhances the Echogenic Properties of ELIP

**Figure 2:**
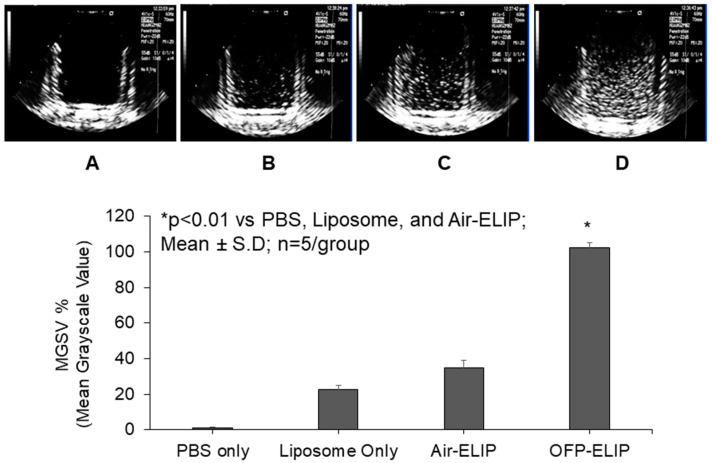
Representative images and echogenicity of liposomes at 4 μg/mL with/without gas. (**A**) PBS only; (**B**) liposomes only; (**C**) air-containing ELIP loaded with CRISPR complex. (**D**) Octafluoropropane (OFP)-containing ELIP loaded with CRISPR complex.

The ability of echogenic liposomes to generate cavitation nuclei under ultrasound stimulation is a critical characteristic of the system. By encapsulating gases, echogenic liposomes (ELIP) create a structure with gas and therapeutic co-encapsulation in a signal carrier, resulting in both improved imaging contrast and ultrasound-enhanced therapeutic delivery properties. In research and clinical applications, the echogenicity ensures the ELIP is traceable and contents releasable upon ultrasound stimulation [16,21,26]. To explore and optimize the impact of different gases on ELIP sensitivity to ultrasound-triggered release, octafluoropropane (OFP) or air was loaded into 5 mg/mL liposomes consisting of MC3/DODAP/DSPC/cholesterol/PEG2000 PE (45:10:9:34.6:1.4, mole ratio) at the same gas pressure using the freezing-thawing/lyophilizing method. The echogenicity of liposomes with or without gas was then measured and compared. Echogenicity measurements revealed that 4 µg/mL OFP-containing liposomes displayed over a 2-fold increase in echogenicity compared to air-containing or liposome-only samples (Figure 2). These findings suggest that OFP-loaded echogenic liposomes are more responsive to ultrasound, leading to more efficient release of contents compared to air-containing samples, indicating their potential for enhanced ELIP performance. Therefore, subsequent experiments were performed on echogenic liposomes containing OFP.

### 3.3. Enhanced Delivery of NF-κB-FITC to Cultured Mouse Neonatal Cardiomyocytes In Vitro and Rat Heart In Vivo Using ELIP and Ultrasound

The ELIP structure allows for both hydrophilic and hydrophobic therapeutic agents to be incorporated into the vesicles, making them suitable for a wide range of gene and drug therapies. Its structure also shields these agents from environmental factors, thereby preventing degradation or elimination before reaching the disease site and ensuring effectiveness [11,12,13,14,23,24,25]. Our research has focused on harnessing ultrasound to enhance the delivery of ODN in cultured neonatal cardiomyocytes in vitro and rat hearts in vivo through the utilization of the ELIP.

**Figure 3:**
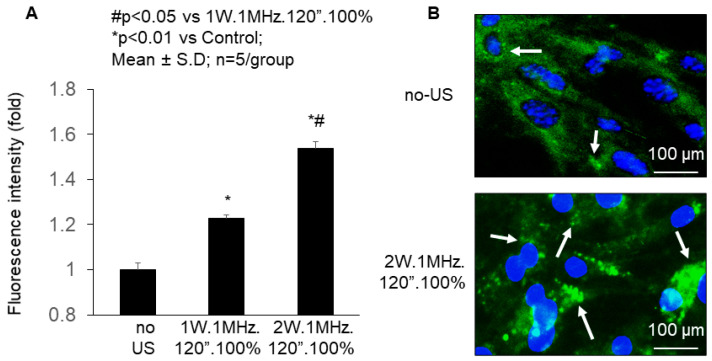
Dose–response (**A**) and fluorescent imaging (**B**) of Ultrasound/NFκB-FITC-ELIP enhanced transfection efficiency into neonatal C57BL/6J mouse cardiomyocytes in vitro. Cardiomyocytes were cultured and then split. (**A**) Following, the cardiomyocytes suspended with PBS were treated with ELIP-NF-κB-FITC (final concentration 10 nM) with different ultrasound parameters, and then the cardiomyocytes were washed with PBS. The fluorescent intensity of the cardiomyocytes was measured using a Synergy microplate reader. Excitation and emission monochromators were set at 490 and 520 nm, respectively. (**B**) Representative fluorescent images of the cardiomyocytes were shown. US: ultrasound.

A fluorescein isothiocyanate-labeled decoy ODN with high affinity for the nuclear factor kappa B (NF-κB-FITC) was used to test the efficacy of ELIP at delivering short nucleic acid into cardiomyocytes. Specifically, our study investigated the dose–response relationship of ultrasound/NF-κB-FITC-ELIP in the transfection efficiency of murine cardiomyocytes in vitro. To achieve this, we isolated cardiomyocytes from neonatal C57BL/6J mice and treated them with NF-κB-FITC-ELIP, followed by ultrasound application with varying parameters. Quantitatively, ultrasound (Intensity: 2 W/cm^2^. Frequency: 1 MHz/cm^2^. Duration: 120 s. Duty cycle: 100%) provided a significant increase (1.5 ± 0.03 fold changes, *p* < 0.01 vs. others) in mean green fluorescence intensity (Figure 3A). Representative fluorescent images of ultrasound/NF-κB-FITC-ELIP substantiated the higher transfection efficiency in the cardiomyocytes (Figure 3B). These findings strongly indicate that ELIP has the potential to release the loaded nucleic acids and enhance transfection efficiency in cardiomyocytes by modulating ultrasound parameters in vitro.

**Figure 4:**
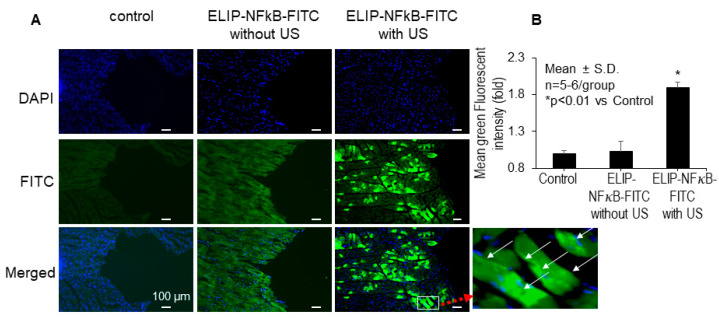
Representative images (**A**) and quantitation (**B**) of fluorescent intensity were shown in rats in vivo. DAPI nuclear staining (blue), FITC immunofluorescence, and a merged image are shown in panels, respectively, using the Nikon H600L imaging System. US: ultrasound.

Beyond our in vitro investigation, our research extended to evaluating the efficacy of ultrasound in conjunction with ELIP for gene delivery in vivo. We administered NF-κB-FITC-ELIP, with or without ultrasound, to rats via left ventricle injection with aortic clamping. The data showed that ultrasound, in combination with ELIP, enhanced the penetration of NFκB-FITC ODN from the endocardium through the ventricular walls (Figure 4A). Quantitatively, ultrasound provided a 2-fold increase (2.0 ± 0.27, *p* < 0.01 vs. no ultrasound) in mean green fluorescence intensity (Figure 4B). These results indicate that ultrasound application in combination with ELIP can enhance the efficacy of gene delivery in vivo.

### 3.4. Characterization of ELIP-CRISPR Complex

**Figure 5:**
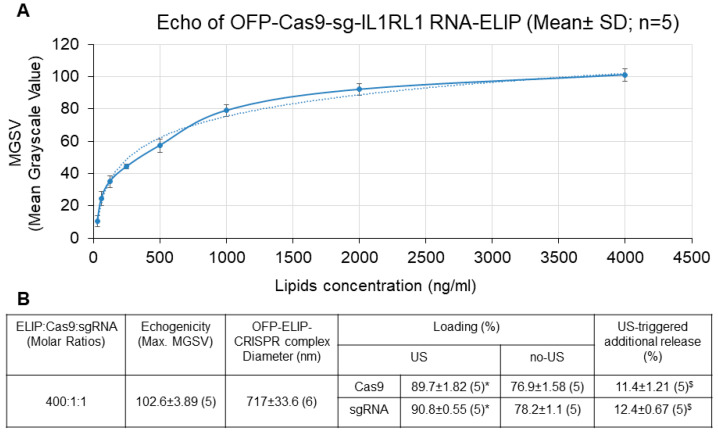
Characterization of OFP-ELIP-CRISPR complex. (**A**). Dose-dependent response in echogenicity (maximum mean grayscale value). Dashed line: trendline (logarithmic). (**B**). Loading efficiency and ultrasound-triggered release of Cas9 and sg-IL1RL1 RNA from OFP-ELIP at 4 μg/mL of ELIP. US: Ultrasound (1 MHz, 1 W/cm^2^, 100%, 15″ for loading or 120″ for release). * *p* < 0.001, as compared to no-US. $ *p* < 0.01, as compared to baseline. Mean ± SD (*n*).

The Echogenic Liposomes (ELIP) have the capability to transport genetic or therapeutic agents. Ultrasound application to specific locations can enhance drug delivery efficiency [10,11,12,13,14,20]. Understanding and harnessing the echogenic properties of ELIP structure aid in advancing drug delivery systems. The characterization of the ELIP-CRISPR complex in terms of echogenicity (ultrasound reflectivity), size, loading efficiency, and ultrasound-triggered release was evaluated. As shown in Figure 5A, the ELIP-CRISPR complex exhibited a concentration-dependent increase in echogenicity, correlating with ELIP concentrations ranging from 0.031 μg/mL to 4 μg/mL. This increase was evidenced by the rise in mean grayscale value (MGSV) from 11.9 ± 3.16 to 102.6 ± 3.89. These findings indicate a favorable mean grayscale value (MGSV = 102.6 ± 3.89) at an ELIP concentration of 4 μg/mL. As shown in Figure 5B, the application of ultrasound enhanced loading efficiency compared to scenarios without its application and triggered an additional release of 11% and 12% of the entrapped Cas9 and sgRNA, respectively, indicating a positive response of the ELIP to ultrasound stimulation. Beckman Coulter Multisizer 4 analysis enabled the determination of both the numbers and size characteristics of ELIPs. Its diameter ranged from 685 to 752 nm, with corresponding numbers ranging from 4.2 × 10^9^ to 4.8 × 10^9^ liposomes/mg lipid. In addition, these results demonstrate the consistency between the amounts of Cas9 and sgRNA encapsulated within the ELIP at the molar ratio of ELIP: Cas9: sgRNA. These findings suggest that the ELIP represents a promising avenue for delivering CRISPR-based genetic materials and facilitating ultrasound-induced release at disease sites.

### 3.5. Therapeutic Efficiency in Terms of Gene Disruption of Cas9/sg-IL1RL1 RNA Components

Given that such delivery methods are particularly suitable for ODN in neonatal cardiomyocytes, ultrasound application in combination with ELIP enhanced payload uptake and subsequent biological effects in the heart. This platform offers an exciting avenue for research in CRISPR-based gene therapy for heart diseases, such as cardiomyopathies caused by gene mutations and changes in expression.

**Figure 6:**
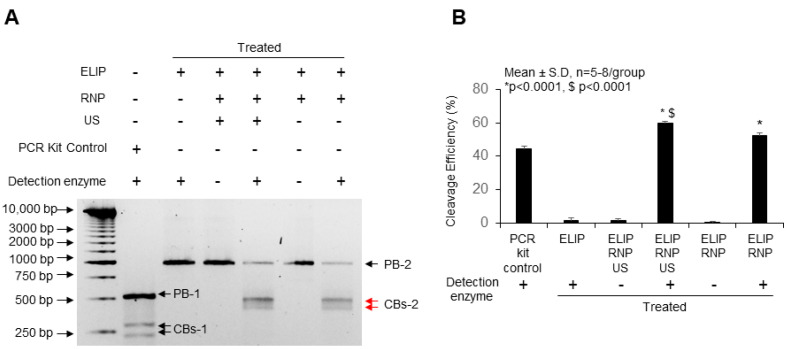
Efficiency of Cas9/sg-IL1RL1 RNA components for gene disruption in mouse neonatal cardiomyocytes using the ELIP-CRISPR delivery platform. Cardiomyocytes were transfected with GeneArt^®^ CRISPR All-In-One vectors targeting *Mus musculus* IL1RL1 locus regions using ELIP. PCR amplification with flanking primers was followed by re-annealing, treatment with Detection Enzyme, and electrophoresis on a 2% agarose gel. (**A**) Representative gel images from the Genomic Cleavage Detection Assay. (**B**) Significance was determined using one-way analysis of variance (ANOVA) with Tukey’s posthoc test (* *p* < 0.0001). $ *p* < 0.0001 compared to ELIP + RNP without ultrasound (US). RNP: Cas9/sg-IL1RL1 RNA ribonucleoprotein complex. PB: Parental Band, PB-1: 516 bp, PB-2: 976 bp. CBs: Cleaved Bands, CBs-1: 225 bp and 291 bp, CBs-2: 478 bp and 498 bp. PCR kit control: Control Template and Primers. DNA Ladder: 1 Kb Plus DNA Ladder (Accuris SmartCheck).

Isolated cardiomyocytes were transfected with Cas9/sg-IL1RL1 RNA RNP using the ELIP-CRISPR delivery platform, and genomic DNA was subsequently analyzed to quantify target gene-specific double-strand breaks (Figure 6). Compared to cardiomyocytes treated with ELIP alone, the ELIP-Cas9/sg-IL1RL1 RNA treatment led to an increase in cleavage efficiency (52.3 ± 1.59%). Cleavage efficiency further increased (59.6 ± 1.37%) in ELIP-Cas9/sg-IL1RL1 RNA treatment with ultrasound exposure. Two cleaved bands of 478 and 498 bp in size were observed. In this work, we demonstrate that our ELIP-CRISPR delivery platform is versatile in its ability to deliver CRISPR/Cas9 complex such as Cas9/sgRNA RNP. Interestingly, in vitro, our ELIP-Cas9/gRNA produced gene cleavage efficiencies of 59% and 52% with and without ultrasound, respectively, as assessed using the GeneArt Genomic Cleavage Detection assay. This is far superior to the 30% cleavage efficiency obtained using Lipofectamine CRISPRMax with Cas9/gRNA RNP under Surveyor assay analysis, as reported by Gong et al. [22]. In our upcoming research, we will utilize labeled sgRNA molecules as an indicator of efficient delivery. This approach of integrating labeled sgRNA molecules into the construction of a CRISPR/Cas9 delivery system presents numerous advantages, including the ability to visualize and monitor delivery processes, optimize conditions, and ensure the reliability of the system for precise genome editing.

## 4. Discussion

CRISPR gene editing is a promising gene therapy approach, although the development of efficient delivery strategies is fundamental for its success. Gene therapy offers substantial potential in addressing defective genes linked to hypertrophic cardiomyopathy and Duchenne muscular dystrophy by utilizing AAV technology [27,28]. Considering concerns related to viral immune responses and associated risks, non-viral vectors are regarded as a safer alternative, known for their ability to accommodate larger payloads [1,3,13,29,30,31]. Through the integration of positively charged lipids, we’ve engineered acoustically responsive liposomes, facilitating the incorporation of CRISPR-based genetic material inside the lipid vesicle and the release of this cargo at target tissue sites. The superiority of the ELIP as a CRISPR-based material delivery system can be attributed to three primary factors: (i) an enhanced loading efficiency of CRISPR-based genetic material in response to ultrasound application, (ii) the additional release of the CRISPR-based material from the gas-containing echogenic liposomes (ELIP) triggered by ultrasound, and (iii) an enhanced uptake of the released payloads by the cells. To optimize this effect, precise adjustments in lipid selection, RNP preparation, ultrasound settings, and cytotoxicity assessment are crucial. Key steps include ensuring high-quality RNP purification, optimizing lipid-to-CRISPR ratios and concentrations for efficient genome editing, and experimenting to identify the optimal ultrasound parameters based on cell type and liposomal formulation. These measures contribute to improved loading and gene editing efficiencies in gas-containing liposomes for CRISPR applications.

The incorporation of certain gases within liposomes, such as OFP in the present case, enhances the echogenic properties of the lipid vesicles, thereby improving controlled release and increasing the concentration of CRISPR-based genetic material within liposomes. Positively charged lipids and ultrasound-triggered events optimize loading, release, and cellular uptake. Ultrasound-responsive liposomes facilitate endosomal fusion, enabling cytosolic release from endosomes through various mechanisms like microbubble oscillation. By promoting the escape of payloads from endosomes, ultrasound enhances the overall therapeutic efficacy of the delivery system. This is particularly important for CRISPR-based genetic material that needs to reach specific intracellular compartments to modulate gene expression or cellular functions. For example, in the case of mRNA (messenger RNA), the targeted intracellular compartment is typically the cytosol, where these molecules can exert their influence on gene expression. On the other hand, for DNA delivery, the payload needs to reach the nucleus for various gene-editing or gene-modifying applications. The ultrasound-controlled release can be crucial for minimizing off-target effects and ensuring that the therapeutic agents are released precisely to the disease site, where precise delivery of genetic material is essential for therapeutic success. In summary, this approach aims to improve the efficiency and precision of therapeutic payload delivery, enhancing the overall therapeutic effect of heart disease treatment. The combined application of ELIP and ultrasound augments the effectiveness of in vivo CRISPR-based gene editing. Gas-containing liposomes have demonstrated the capacity to amplify cell uptake and release payloads when exposed to ultrasound owing to acoustic cavitation. When ultrasound waves interact with these gas-containing liposomes, they stimulate oscillations, resulting in the creation and collapse of microbubbles. These microbubbles create mechanical forces, including microstreaming and micro-jetting, which can temporarily disrupt cell membranes, thereby facilitating enhanced cellular uptake of the liposomes and efficient release of the payload at the disease site [32,33]. This process promotes the uptake of genetic material into cells and enables controlled release, making it an effective method for targeted drug or gene delivery in disease tissues. Moreover, localized ultrasound-triggered release of payloads may enhance cellular uptakes more effectively than alternative methods. This platform may exhibit fewer adverse effects compared to invasive methods, as lower-power ultrasound is generally considered safe for cells and tissues.

**Figure 7:**
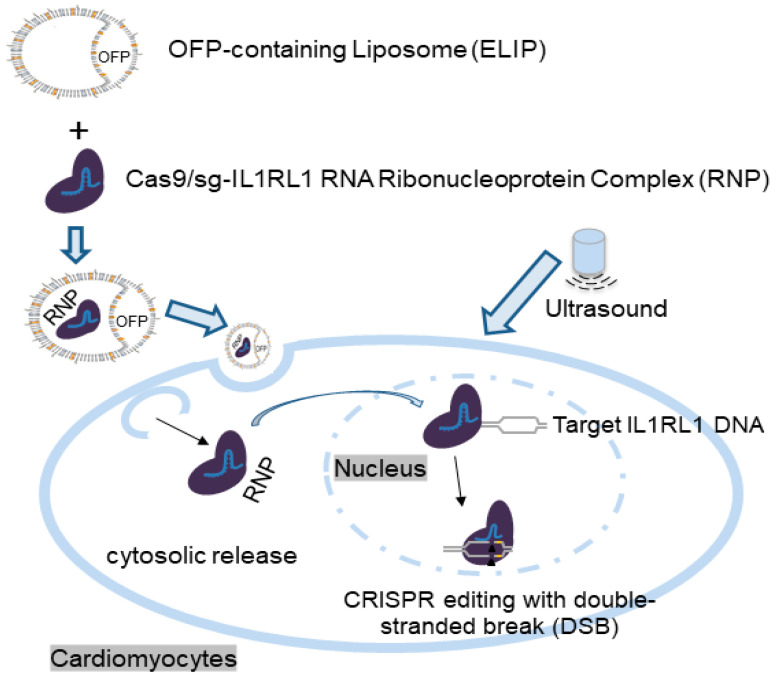
Schematic illustration of the ELIP-based CRISPR delivery platform designed for gene therapy targeting heart disease. In this study, we developed this platform to efficiently deliver Cas9-sg-IL1RL1 RNA RNP to cardiomyocytes, demonstrating an effective approach for CRISPR-based gene editing.

Although several papers have been published on the simultaneous application of ultrasound and gas-containing liposomes to enhance the release of genetic material from nanoparticles and enhance cellular uptake [16,34,35,36,37,38,39], only a limited number of studies have specifically investigated the simultaneous application of delivering CRISPR-based payloads to disease sites for disease treatment [15,16]. Building upon these findings, we have developed an ELIP-CRISPR platform for ultrasound-controlled CRISPR complex delivery, focusing on its potential therapeutic impact on heart disease. By designing consistent and uniform liposomes, this platform provides comparable loading of Cas9 and gRNA. This allows for a balanced incorporation of components, potentially leading to a reliable and balanced gene therapy system. Upon ultrasound stimulation, the ELIP releases both Cas9 and gRNA efficiently, demonstrating the effectiveness and consistency of the preparation process. As shown in Figure 7, we hypothesized that an ELIP-based CRISPR delivery system efficiently transported the CRISPR complex to cardiomyocytes and heart tissue by enhancing loading and penetration upon ultrasound application. The liposomes merge with cell membranes via endocytosis, releasing genetic material into the cytoplasm. The resulting Cas9/gRNA ribonucleoprotein enters the nucleus, facilitating precise genome editing [13]. These experiments showed positive and compatible results, indicating a dependable delivery system for CRISPR-based gene therapy in heart disease. An efficient ELIP-CRISPR complex delivery system could directly target and edit genes linked to heart disease, potentially improving patient outcomes. By modifying genes associated with heart disease, this approach aims to reduce disease susceptibility, promising more effective gene editing and better cardiovascular outcomes. While CRISPR gene-editing technology holds potential for treating heart disease by targeting disease-related genes, there are several challenges to address. More stable and adaptable liposomes with enhanced resistance to various conditions will have to be generated using careful optimization of ultrasound parameters and liposome structures. Advancements in CRISPR technology are expected, emphasizing more efficient and specific components for cardiovascular disease gene therapy. However, extensive preclinical testing is necessary before echogenic liposomes can be considered a safe drug delivery method for CRISPR-mediated cardiovascular gene therapy. Delivering CRISPR complex to target cells and tissues remains challenging due to tissue accessibility.

## 5. Conclusions

Our study indicates that ultrasound-triggered liposomal delivery systems hold promise for gene therapy in heart disease by facilitating gene mutation or alteration. Further research will aim to optimize this delivery system and explore its therapeutic potential in the area of CRISPR technology. Additional research involving animal studies and subsequent clinical trials is vital to assess feasibility and safety. Considering the versatility of ELIP conferred via endless modification of their lipid and gas compositions, this approach represents an exciting and rapidly evolving area of gene therapy research in cardiovascular disease.

## Figures and Tables

**Table 1:** Sequences of single guide RNAs tested in this study (Genome: *Mus musculus*, Gene: IL1RL1 (interleukin 1 receptor-like 1).

Target	Sequence
IL1RL1-targeting sgRNA1	ACUUGUAGGUAAAUCGUCCU
IL1RL1-targeting sgRNA2	CUUGUAGGUAAAUCGUCCUG
IL1RL1-targeting sgRNA3	AGGUAAAUCGUCCUGGGGUC
IL1RL1-targeting sgRNA4	AGCCUCAUUUUCCAGACCCC

**Table 2:** Primer sequences for PCR analysis.

Target	Primers (Amplicon Size: 976 bp)
IL1RL1	fwd: CCGACAAGCAGTCTCTAAGTTC
IL1RL1	rev: CGGCTCATTCCCTTCCTAATC

## Data Availability

The data presented in this study are available on request from the corresponding author. All data generated or analyzed during this study are included in the manuscript.
